# Serum irisin levels in women with polycystic ovary syndrome: a cross-sectional case-control study

**DOI:** 10.1590/1516-3180.2026.3566.08042026

**Published:** 2026-08-07

**Authors:** Atahan Toyran, Yagmur Minareci, Cavidan Gulerman

**Affiliations:** I Department of Gynecology and Obstetrics, Division of Gynecologic Oncology, Istanbul University, Faculty of Medicine, Istanbul, Türkiye.; II Department of Gynecology and Obstetrics, Division of Gynecologic Oncology, Istanbul University, Faculty of Medicine, Istanbul, Türkiye.; III Clinic of Infertility and Reproductive Medicine, Ankara City Hospital; University of Health Sciences Türkiye, Ankara, Türkiye.

**Keywords:** Polycystic Ovary Syndrome, Myokines, Biomarkers, ROC Curve, Insulin Resistance, Hyperandrogenism, Adiposity, Rotterdam Criteria, Diagnostic Threshold

## Abstract

**BACKGROUND:**

Polycystic ovary syndrome (PCOS) is a common endocrine–metabolic disorder. Irisin is an exercise-related myokine involved in energy homeostasis; however, prior studies have reported inconsistent associations between irisin and PCOS.

**OBJECTIVES:**

To compare serum irisin levels between women with PCOS and non-PCOS controls and to assess the discriminatory performance of irisin for PCOS.

**DESIGN AND SETTING:**

Cross-sectional, case–control study conducted at a tertiary referral center.

**METHODS:**

We enrolled 144 women, 72 with PCOS and 72 non-PCOS as controls. Serum irisin levels were measured using an enzyme-linked immunosorbent assay. Adjusted comparisons were performed using a general linear model, and the association between irisin and PCOS was further evaluated using multivariable binary logistic regression. Receiver operating characteristic (ROC) analysis was used to assess the diagnostic performance of irisin.

**RESULTS:**

Serum irisin levels in the PCOS group were significantly lower than those in the control group (p = 0.006). In the age and body mass index (BMI) adjusted ANCOVA model, PCOS status remained significantly associated with lower log-transformed irisin (p = 0.002). In a multivariable logistic regression analysis adjusted for age and BMI, log-transformed irisin levels remained independently associated with PCOS (p = 0.006). In ROC analysis, the area under the ROC curve for irisin was 0.632; an irisin cut-off of ≤ 16.2 ng/mL resulted in 87.5% sensitivity and 38.89% specificity.

**CONCLUSIONS:**

Women with PCOS had lower circulating irisin levels than controls, and this association persisted after adjusting for age and BMI. Discriminatory performance was modest, and specificity was low at the selected cutoff. Larger studies are warranted to validate these findings.

## INTRODUCTION

Polycystic ovary syndrome (PCOS) is a prevalent endocrine-metabolic disorder, affecting 5–20% of women of reproductive age depending on the diagnostic criteria used. ^1,2^ The syndrome is characterized by irregular menstruation, hyperandrogenism, and polycystic ovarian morphology.^3^ Insulin resistance is common in PCOS and plays a significant role in its pathophysiology, contributing to increased risks of type 2 diabetes mellitus, metabolic syndrome, and cardiovascular complications.^4,5^

Recent studies have highlighted the role of irisin, a myokine induced by exercise, in regulating energy balance and metabolism. Primarily synthesized in skeletal muscle, irisin promotes the conversion of white to brown adipose tissue, thereby enhancing thermogenesis.^6,7^ In women with PCOS, altered irisin levels may be an adaptive response to insulin resistance; however, findings on irisin levels in patients with PCOS are contradictory.^8,9^

## OBJECTIVE

The primary objective of this study was to determine serum irisin levels in patients with PCOS compared to non-PCOS controls, evaluate irisin’s potential as a diagnostic marker, and examine its relationship with insulin resistance. By proposing a cutoff value through receiver operating characteristic (ROC) analysis, we sought to fill a notable gap in the literature and contribute to the ongoing debate on whether irisin can serve as a clinically useful marker in the management of PCOS.

## METHODS

### Study design and ethical approval

This study was designed as an observational, cross-sectional, case-control investigation. It was approved by the Ankara City Hospital Clinical Research Ethics Committee No. 1 on September 2, 2020 (Approval No. E1–20–1039). Written informed consent was obtained from all participants prior to enrollment.

### Study population and sample size

A total of seventy-two women with PCOS, diagnosed based on the Rotterdam criteria, were consecutively recruited from the infertility/PCOS clinic, while seventy-two non-PCOS women as control were selected from those visiting the reproductive endocrinology clinic for benign conditions. An a priori sample size calculation was performed for the primary outcome (difference in serum irisin levels between PCOS and controls). Based on the between-group difference reported by Abali et al.^3^ (158.5 ± 123.3 vs. 222.9 ± 152.2 ng/mL), the targeted effect corresponded to an expected mean difference of 64.4 ng/mL (Cohen’s d ≈ 0.47). With a two-sided significance level of 0.05 and 80% power (β = 0.20) at a 1:1 allocation ratio, the required sample size was approximately seventy-one participants per group, which was rounded up to seventy-two per group (total n = 144).

The PCOS group included women aged 20–49 years who satisfied at least two of the following three Rotterdam criteria: oligo-anovulation, clinical or biochemical hyperandrogenism, or polycystic ovarian morphology observed by transvaginal ultrasonography (ovarian volume > 10 mL or at least one ovary with ≥ 12 follicles measuring 2–9 mm).^10^ Controls were aged 20–49 years, with regular menstrual cycles, and did not meet the Rotterdam diagnostic criteria for PCOS. Isolated PCOS-related features, such as hirsutism, biochemical hyperandrogenism, or polycystic ovarian morphology, were allowed provided that the full diagnostic criteria for PCOS were not met.

The exclusion criteria were as follows: refusal to participate, age below twenty or above forty-nine years, pregnancy, chronic systemic diseases, cardiovascular risk, liver disease, rheumatologic conditions, recent infections, previous ovarian surgery, hormonal therapy in the previous six months, smoking, and medications affecting insulin metabolism.

### Clinical and biochemical assessments

Menstrual irregularity was defined as cycle length of less than twenty-one or greater than thirty-five days,^1^ and hirsutism was assessed using the modified Ferriman–Gallwey score, with a score of ≥ 8. Biochemical hyperandrogenism was defined as total testosterone > 59 ng/dL, androstenedione > 15 nmol/L, free androgen index > 5, or free testosterone > 4.2 pg/mL.^11^ Insulin resistance was calculated using the Homeostatic Model Assessment of Insulin Resistance (HOMA-IR) formula (fasting glucose [mg/dL] × fasting insulin [mIU/mL]/405), with HOMA-IR ≥ 3.2 considered indicative of insulin resistance.^12^ Definitions for dyslipidemia and body mass index (BMI) classifications followed the American Diabetes Association, World Health Organization, and established guidelines.^13,14,15^

### Biochemical measurements

Fasting blood samples (10 mL) were collected on the second or third day of each participant’s menstrual cycle. Samples were centrifuged at 4,500 rpm for 15 minutes, and the resulting serum was stored at −80°C until analysis. Serum irisin concentrations were quantified using an enzyme-linked immunosorbent assay (ELISA) kit (Human Irisin ELISA, Sunred Biological Technology., China, Catalog No. 201–12–5328) with a detectable concentration range of 0.2–60 ng/mL. The sensitivity (limit of detection) of the assay was 0.157 ng/mL.

Assay validation was conducted using standard curve measurements. The intra-assay coefficient of variation (CV) ranged from 0% to 8% for low to medium concentrations and up to 16% for higher concentrations. The inter-assay CV was within an acceptable range, ensuring assay reliability. All measurements were performed in duplicate, and optical density (OD) readings were obtained at 450 nm. The precision of the assay depended on the position of the measurement on the dose-response curve, and calibration was conducted using seven standards ranging from 0 to 64 ng/mL.

Additional parameters, including total testosterone, DHEA-S, SHBG, HbA1c, fasting glucose, fasting insulin, 25-hydroxy vitamin D, FSH, LH, estradiol, AMH, and lipid profiles, were obtained from blood samples collected on the same day as serum irisin sampling and were retrieved from hospital records.

### Statistical analysis

Statistical analyses were performed using NCSS (NCSS LLC, Utah) and IBM SPSS Statistics (IBM Corp., Armonk, NY, USA). Shapiro–Wilk and box plot analyses assessed normality. For normally distributed variables, group comparisons utilized Student’s t-test; non-normally distributed variables were assessed with the Mann–Whitney U test. Chi-square, Fisher’s Exact, and Fisher-Freeman-Halton tests compared categorical data. Because serum irisin values were right-skewed, they were log-transformed prior to multivariable modeling. To address potential confounding by age and BMI, adjusted group differences in log-transformed irisin were assessed using a general linear model (ANCOVA) with age and BMI as covariates; homogeneity of variances was evaluated using Levene’s test. In addition, a multivariable binary logistic regression model was used to examine the association between log-transformed irisin and PCOS after adjustment for age and BMI. Results were reported as adjusted estimates and odds ratios (OR) with 95% confidence intervals (95% CI). ROC curve analysis was used to evaluate the discriminatory performance of irisin for PCOS, reporting the area under the curve (AUC), sensitivity, specificity, and the selected cutoff value. All tests were two-sided, and p < 0.05 was considered statistically significant.

## RESULTS

Comparisons of demographic, anthropometric, biochemical, and hormonal parameters between the PCOS and control groups are shown in **Table 1 and 2**. The mean age was significantly lower in the PCOS group (24.01 vs. 28.22 years; p = 0.001), and BMI was higher (p = 0.002). The PCOS group had a significantly higher LH/FSH ratio, total testosterone, DHEA-S, and free androgen index (all p < 0.01) and lower SHBG levels (p = 0.001).

**Table 1:** Comparison of demographic, anthropometric and metabolic parameters between the PCOS and non-PCOS control groups

	PCOS (n = 72) *Mean* ± *SD*	Non-PCOS Control (n = 72) *Mean* ± *SD*	*p*
Age *(years)*	24.01 ± 3.66	28.22 ± 4.65	***0.001***
BMI *(kg/m^2^)*	27.02 ± 6.11	24.04 ± 5.31	***0.002***
Fasting Blood Glucose *(mg/dL)*	88.54 ± 9.19	87.42 ± 10.22	*0.382*
HbA1c (%)	5.5 ± 0.4	5.60 ± 1.2	*0.692*
Fasting Insulin *(mu/L*)	23.47 ± 37.39	13.23 ± 11.2	***0.001***
HOMA-IR	4.39 ± 3.53	3.2 ± 3.02	***0.003***
Total Cholesterol *(mg/dL)*	160.46 ± 27.18	168.09 ± 45.2	*0.171*
LDL *(mg/dL)*	89.73 ± 24.78	92.03 ± 27.5	*0.645*
HDL *(mg/dL)*	46.35 ± 12.49	58.46 ± 16.2	***0.001***
Triglycerides *(mg/dL)*	112.54 ± 57.93	120.16 ± 128.96	*0.107*
25(OH) Vitamin D *(ng/mL)*	14.89 ± 13.02	15.87 ± 7.78	*0.103*

Abbreviations: BMI, Body Mass Index; PCOS, Polycystic Ovary Syndrome; LDL, Low-Density Lipoprotein; HDL, High-Density Lipoprotein; HOMA-IR, Homeostasis Model Assessment of Insulin Resistance.

**Table 2:** Comparison of hormonal parameters and Polycystic Ovary Syndrome-related clinical features between the PCOS and non-PCOS control groups

		PCOS (n = 72)	Non-PCOS Control (n = 72)	*p*
**Hormonal Parameters**		*Mean* ± *SD*	*Mean* ± *SD*	
*Anti-Müllerian Hormone (*μ*g/L)*		6.39 ± 2.43	3.76 ± 2.53	***0.001***
*Total Testosterone (ng/dL)*		38.97 ± 11.38	31.10 ± 13.08	***0.001***
*LH/FSH ratio*		1.39 ± 0.85	1.04 ± 0.75	***0.008***
*SHBG (nmol/L)*		35.81 ± 20.02	66.22 ± 39.5	***0.001***
*DHEA-S (*μ*g/dL)*		262.66 ± 110.25	214.15 ± 100.01	***0.007***
*Free Androgen Index (FAI)*		5.46 ± 4.42	2.36 ± 1.81	***0.001***
PCOS-related Clinical Features* (n(%))				
*Hirsutism*	Absent	8 (11.1)	67 (93.1)	***0.001***
	Present	64 (88.9)	5 (6.9)	
*Biochemical Hyperandrogenism*	Absent	45 (62.5)	53 (73.6)	*0.153*
	Present	27 (37.5)	19 (26.4)	
*Menstrual Irregularity*	Absent	7 (9.7)	72 (100)	***0.001***
	Present	65 (90.3)	0 (0)	
*Polycystic Ovarian Morphology*	Absent	6 (8.3)	68 (94.4)	***0.001***
	Present	66 (91.7)	4 (5.6)	

* Control participants could have isolated PCOS-related features; however, none fulfilled the Rotterdam diagnostic criteria for PCOS.

Abbreviations: PCOS, Polycystic Ovary Syndrome; LH, Luteinizing Hormone; FSH, Follicle Stimulating Hormone; SHBG, Sex Hormone-Binding Globulin; DHEA-S, Dehydroepiandrosterone Sulfate; FAI, Free Androgen Index; AMH, Anti-Müllerian Hormone.

The PCOS group showed higher fasting insulin and HOMA-IR levels (p = 0.001; p = 0.003) and a higher mean AMH level (6.39 μg/L versus 3.76 μg/L, p = 0.001). HDL was significantly lower in the PCOS group (46.35 mg/dL versus 58.46 mg/dL, p = 0.001). Hirsutism, menstrual irregularity, and polycystic ovarian morphology were significantly more prevalent in the PCOS group (all p = 0.001); hirsutism was observed in 88.9%, menstrual irregularity in 90.3%, and polycystic morphology in 91.7% of the PCOS group.

**Figure 1:**
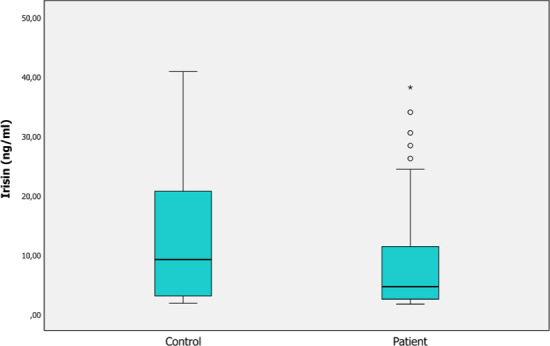
Distribution of Irisin Levels in PCOS and non-PCOS Control Groups

Serum irisin levels were significantly lower in the PCOS group (median, 4.61 ng/mL) than in the control group (median, 9.18 ng/mL; p = 0.006). **Table 3** and **Figure 1** show irisin levels in both groups.

**Table 3:** Evaluation of irisin levels in the PCOS and non-PCOS control groups

		PCOS (n = 72)	Non-PCOS Control (n = 72)	*p*
Irisin Level (ng/mL)	*Median (Q1–Q3)*	4.61 (2.5–11.38)	9.18 (3.04–20.67)	***0.006***

Abbreviations: PCOS: Polycystic Ovary Syndrome

Values are presented as median (IQR). Groups were compared using the Mann–Whitney U test.

In unadjusted analyses, no statistically significant association was found between age and irisin levels (p > 0.05). However, a weak but statistically significant negative correlation was observed between BMI and irisin levels (ρ = −0.299; p = 0.001) and between body weight and irisin levels (ρ = −0.268; p = 0.001), indicating that irisin levels decreased as BMI and body weight increased.

Given the between-group differences in age and BMI and the observed inverse association between BMI and irisin, additional multivariable analyses were performed using natural log-transformed irisin values. In ANCOVA adjusting for age and BMI, PCOS status remained significantly associated with lower log-transformed irisin (F (1,140) = 10.338, p = 0.002; partial η^2^ = 0.069). At the mean covariate values (age = 26.12 years; BMI = 25.53 kg/m^2^), the estimated marginal mean of log-transformed irisin was 2.181 (95% CI, 1.953–2.409) in controls and 1.619 (95% CI, 1.391–1.847) in the PCOS group, corresponding to an adjusted mean difference of 0.562 (95% CI, 0.216–0.908; p = 0.002). In the same model, age (p = 0.002) and BMI (p = 0.048) were also associated with log-transformed irisin, and homogeneity of variances was not violated (Levene’s p = 0.278).

In the multivariable binary logistic regression including log-transformed irisin, age, and BMI, log-transformed irisin remained independently associated with PCOS status (B = −0.638, p = 0.006; OR = 0.529, 95% CI, 0.336–0.831). BMI was positively associated with PCOS (B = 0.083, p = 0.033; OR = 1.086, 95% CI, 1.007–1.172), whereas age showed an inverse association (B = −0.284, p < 0.001; OR = 0.753, 95% CI, 0.676–0.839). The overall model was significant (χ^2^(3) = 51.645, p < 0.001; Nagelkerke R^2^ = 0.402) and correctly classified 75.7% of participants (77.8% of the non-PCOS controls and 73.6% of the PCOS group).

**Figure 2:**
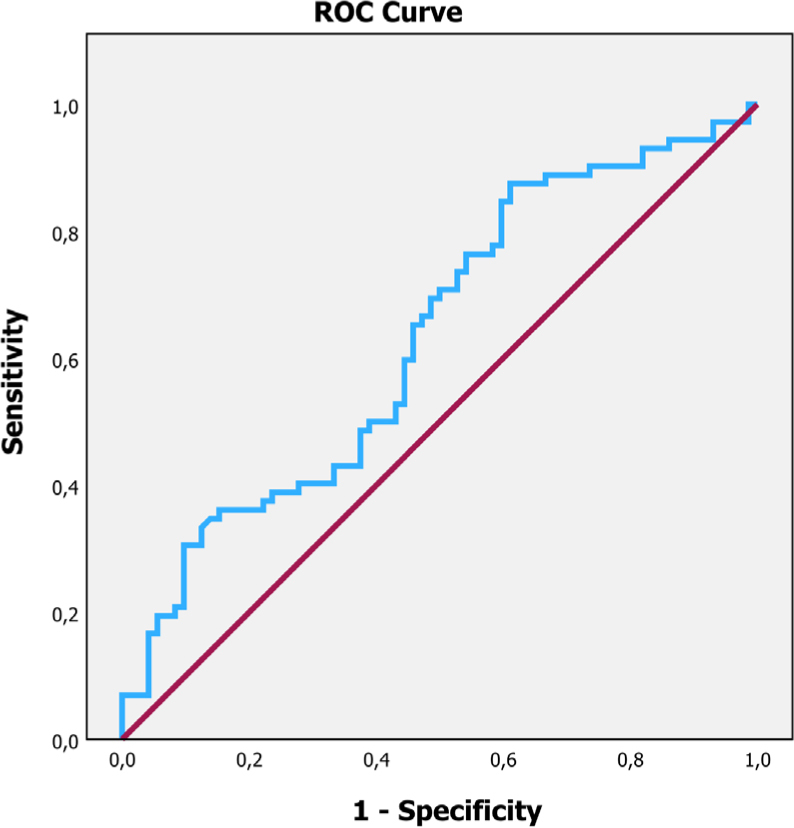
Receiver Operating Characteristic Curve Graph for Irisin by Groups.

ROC analysis was performed to determine a cutoff value for irisin in the diagnosis of PCOS, based on the significant difference in irisin levels between the PCOS and control groups. The optimal cutoff value was calculated as 16.2 ng/mL, with a sensitivity of 87.5% and a specificity of 38.89%. The positive predictive value was 58.9%, and the negative predictive value was 75.7%. The AUC was 0.632 (95% CI, 0.541–0.723, standard error: 0.046), and the association was statistically significant (p = 0.006). Individuals with irisin levels of 16.2 ng/mL or lower had a 4.45-fold higher odds of PCOS (95% CI, 1.91–10.36) (**Table 4, Figure 2**).

**Table 4:** Diagnostic screening tests and Receiver operating characteristic curve results for irisin by group

	Diagnostic Screening					ROC Curve		*p*
	Cut-off	Sensitivity (%)	Specificity (%)	Positive Predictive Value (%)	Negative Predictive Value (%)	Area	95% CI	
Irisin	≤ *16.2*	87.5	38.89	58.9	75.70	0.632	0.541–0.723	***0.006***

Abbreviations: ROC, Receiver Operating Characteristic.

Among all participants (n = 144), significant but weak correlations were found between irisin levels and HOMA-IR (negative, ρ = −0.184; p = 0.038), 25(OH) vitamin D (positive, ρ = 0.185; p = 0.042), SHBG (positive, ρ = 0.176; p = 0.044), total cholesterol (positive, ρ = 0.190; p = 0.044), and HDL (positive, ρ = 0.271; p = 0.004).

Irisin levels were significantly lower in women with hirsutism (8.16 ng/mL) than in those without (12.5 ng/mL; p = 0.002). Additionally, participants with polycystic ovarian morphology had significantly lower irisin levels (7.19 ng/mL) than those without this feature (13.49 ng/mL; p = 0.002). No statistically significant differences were observed in irisin levels in relation to insulin resistance, biochemical hyperandrogenism, or menstrual irregularity (p > 0.05).

## DISCUSSION

Recent literature has shown increasing interest in investigating irisin levels in patients with PCOS; however, study results remain contradictory.^9^ Given the conflicting evidence on the association between irisin and PCOS, our aim was to evaluate irisin as a candidate biomarker for PCOS and explore its discriminatory performance by establishing an ROC-based cutoff.

Our results demonstrated that irisin levels were significantly lower in the PCOS group than in the control group. Importantly, this difference persisted after adjusting for age and BMI in both the ANCOVA model and multivariable logistic regression. This finding is consistent with the studies by Abali et al.^3^ and Wang et al.,^16^ which reported reduced irisin levels in patients with PCOS. However, conflicting results exist, with some studies documenting elevated irisin levels in PCOS, and others reporting no significant difference.^17,18,19^ These discrepancies may reflect differences in population characteristics, PCOS diagnostic criteria, and laboratory assay methods.

Reduced peroxisome proliferator-activated receptor gamma coactivator 1-alpha (PGC-1α) signaling, which regulates fibronectin type III domain-containing protein 5 (FNDC5) transcription in skeletal muscle, may explain the lower irisin levels observed in women with PCOS.^20^ Although we did not directly assess PGC-1α/FNDC5 in this study, prior evidence of impaired mitochondrial/oxidative pathways in PCOS provides a plausible biological context for reduced irisin.^21^

Because a higher BMI is observed in women with PCOS,^22^ and because BMI may influence irisin secretion, we also examined the relationship between adiposity and irisin. We observed a significant negative correlation between BMI and irisin levels, suggesting that irisin levels decrease as BMI increases. This finding contrasts with that of Bostanci et al.,^23^ who reported a positive correlation between BMI and irisin levels; however, it aligns with that of Wang et al., who also observed a negative correlation between irisin levels and body weight and BMI.^16^ Similarly, a meta-analysis by Cai et al.^9^ proposed that BMI may modulate irisin via insulin resistance and physical activity. These mixed findings suggest that BMI, adiposity distribution, and lifestyle factors collectively influence circulating irisin.

Regarding insulin resistance, irisin did not significantly correlate with fasting glucose or fasting insulin, diverging from some reports in which higher insulin or glucose levels were correlated with elevated irisin levels.^9,16^ Interestingly, we detected a weak but negative correlation between irisin and HOMA-IR (ρ = −0.184; p = 0.038), unlike Wang et al.^16^ who reported a positive association. Furthermore, we did not observe a significant difference in irisin levels when comparing participants with and without insulin resistance defined by HOMA-IR ≥ 3.2, suggesting that insulin resistance alone may not fully dictate irisin dynamics in PCOS.

In the ROC analysis, irisin showed modest discrimination for PCOS (AUC = 0.632). The selected cut-off (≤ 16.2 ng/mL) provided high sensitivity but low specificity, limiting its value as a standalone diagnostic test. Therefore, this threshold should be considered exploratory requiring external validation in independent cohorts.

Although preclinical studies suggest that irisin may influence metabolic and reproductive pathways, its clinical implications in humans remain unclear.^24,25,26^ Further translational studies are warranted before therapeutic conclusions can be drawn.

Several limitations should be considered when interpreting our findings. First, this was a single-center, cross-sectional case-control study conducted in a tertiary referral setting, which limits generalizability and precludes causal inference. Second, the PCOS and control groups differed in age and BMI and although we addressed these differences using age- and BMI-adjusted models, residual confounding cannot be excluded, particularly from unmeasured factors, such as physical activity, diet, and body composition/fat distribution. These unmeasured factors, together with heterogeneity in irisin assay methods across studies, may partly explain the conflicting results reported in the PCOS literature.

## CONCLUSION

Circulating irisin levels were lower in women with PCOS than in non-PCOS controls, and this association persisted after adjusting for age and BMI. Irisin demonstrated modest discriminatory performance with low specificity at the selected cutoff; therefore, the results should be considered exploratory requiring external validation. Larger, well-characterized cohorts are needed to confirm these findings and clarify whether irisin adds value alongside established clinical and biochemical markers.

## Data Availability

Data supporting the findings of this study are available upon request from the corresponding author, Atahan Toyran.
